# Does Patient Access to Clinical Notes Change Documentation?

**DOI:** 10.3389/fpubh.2020.577896

**Published:** 2020-11-27

**Authors:** Charlotte Blease, John Torous, Maria Hägglund

**Affiliations:** ^1^Division of General Medicine, Beth Israel Deaconess Medical Center and Harvard Medical School, Boston, MA, United States; ^2^Department of Psychiatry, Beth Israel Deaconess Medical Center and Harvard Medical School, Boston, MA, United States; ^3^Department of Women's and Children's Health, Uppsala University, Uppsala, Sweden

**Keywords:** electronic medical records, open notes, clinical documentation and communication, transparency & disclosure, natural language processing, medical ethics

Open, honest, and trustworthy communication is crucial to ensure the effective responses of citizens. Paralleling transparency in the arena of public health are new practice policies that are set to transform the transmission of information at the level of doctors and patients. While patients have legally been entitled to obtain copies of their records for many years, in March 2020 federal legislation in the United States (U.S.) mandated that health providers offer all patients rapid and secure online access to their clinical notes via patient portals (“open notes”) (1). Similar developments are underway in the United Kingdom (U.K.) where in April 2020 it was announced that patients in NHS England will be granted online access, albeit prospectively, to their full general practitioners' notes (2). Worldwide, open notes have already been enacted in more than ten countries including Sweden, Estonia, and Norway (3).

A variety of surveys have been conducted into patients' and doctors' experiences of open notes but much less is understood about the objective changes in documentation that may arise as a result of patient access (4–7). We review current research into open notes including clinicians' reports on how they have modified their notes as a result of implementing the practice. Highlighting the potentially beneficial and harmful effects that different types of documentation changes might have on the therapeutic relationship and on patient outcomes, we argue that more research is needed to investigate objective changes in notes as a result of patient access.

## Patients' and Doctors' Experiences of Open Notes

The overwhelming majority of patients who access their records online report positive experiences (5, 7, 8). Patients describe feeling more in control of their care, enhanced understanding of the rationale for treatments and referrals, better remembering their treatment plans, and doing a better job taking their medications (5, 7–9). Only a small proportion—in one U.S, survey of over 22,000 patients between 3 and 5%—report being very confused or more anxious by what they have read (5). We were not able to find any cases of patient harm caused by sharing notes or legal action taken because of something a patient read. In addition, patients also describe interpersonal benefits of access including feeling better about their clinician after reading their notes, enhanced levels of trust, and strengthened goal-alignment and perceptions of teamwork with providers (5, 10).

How do healthcare clinicians view the practice? While the majority of surveyed physicians consider open notes to be a good idea (5) there does appear to be some variation in attitudes both between medical specialties (2, 11–14) and countries (14, 15). Mental health clinicians, for example, including psychiatrists, in the U.S. and in Sweden appear to be more cautious [(11, 12), p. 2]. In a survey at a medical center in the U.S. Veterans Health Administration nearly one in two mental health clinicians (49% *n* = 98) reported that they would be “pleased” if the practice were discontinued. Some healthcare professionals report negative effects of note sharing including perceptions of heightened patient distress or worry from reading notes [(12), p. 2, (14, 15)]. Finally, while around one third of surveyed clinicians report spending more time writing notes (6, 11) most do not perceive an increase in patient contact or visit times [(5, 11, 12), p. 2, (14)] because of open notes.

## Survey Evidence of Documentation Changes

A major focus of current survey research is the influence of open notes on physicians' documentation practices. In multiple surveys, as a result of patient access to their notes, many clinicians report being more mindful of the words that they use (6, 11). For example, in a recent large-scale survey, the majority of primary care physicians describe adjusting their language to avoid being perceived as critical of patients with around half omitting terms such as “non-compliant,” and “patient denies,” or modifying how they document sensitive clinical, mental or social information (6). In addition, around a quarter (26%, *n* = 61) of U.S. primary care physicians report employing more partnering or encouraging language in their notes (6).

As a result of patient access, physicians also report changing their use of medical terminology, and the level of detail included in their notes. For example, in survey research in Sweden, one in five (22%, *n* = 147) mental health clinicians [(12), p. 2], and two in three (67%, *n* = 43) oncologists (15) admitted writing less candid notes. In the U.S., the majority (69%, *n* = 108) of mental health clinicians report writing fewer details (11), and a quarter (26%, *n* = 63) of primary care physicians report changing how they document differential diagnoses (6). In a major survey of US physicians from different medical specialities, 22% (*n* = 168) did consider their notes less valuable (6) because of open notes.

## Why Documentation Change Matters

Open notes may provide an opportunity to “extend the visit” providing patients more time to read and reflect on their doctors' recommendations away from the pressures and time-restrictions of face-to-face interactions. As a result of patient access, however, the tone and content of clinical notes and any changes to documentation may have the potential to influence the quality of care in both positive and negative ways.

By removing language that may be perceived as negative, or including reassuring or supportive wording, documentation changes may heighten patient perceptions of empathy and strengthen trust in clinicians, factors that are associated with improved patient outcomes, beneficial health behaviors, and increased patient satisfaction (16, 17). In addition, patient recall and understanding of information communicated during visits is often poor (18). Convenient electronic access to clinical notes and changes to documentation that improve the clarity of the notes—for example, using plain language to provide brief but understandable explanations for tests and treatments—may boost patient comprehension, recall, and adherence to medications and care plans.

Results of clinician surveys suggest, however, that there may be some risks to documentation practices of open notes. Knowledge of patient access may create a tension between writing understandable notes balanced against over-simplifying medical information and thereby devaluing the utility of documentation for health professionals. Indeed, medical records can be understood as a form of “cognitive scaffolding” helping to aid physician memory, and facilitate diagnostic reasoning. Well-intentioned strategies by physicians to avert worry or anxiety among patients by failing to list differential diagnoses may undermine these crucial functions of record-keeping. In Norway, healthcare professionals report keeping a “shadow record” to document information that they believe should be inaccessible to the patient, a work-around that may risk patient safety and security (19).

## Measuring Documentation Changes

Although survey research provides valuable insights into physicians' perceptions about changes to documentation, these findings are dependent on self-report and it is not known how response biases affect results. Addressing these challenges, preliminary investigations have sought to analyse the possible objective changes to documentation.

Researchers have examined whether open notes might influence the socio-emotional tone of notes using the computer program the Linguistic Inquiry and Word Count (“LIWC”) (13). The LIWC software has been used extensively in clinical psychology to assess patterns of language use and association with behavior (20). It can be used to track the use of pronouns (such as “we” which is associated with perceptions of partnership); the inclusion of cognitive words (for e.g., “because,” “reason,” “think”); and the use of positive and negative emotion words. Applying this program to investigate changes in clinical notes in oncology, investigators reported no significant modification of the linguistic character of documentation pre- and post-implementation of open notes (13). Although a promising method of assessing changes in collaborative language, this approach has important limitations. While the LIWC can quantify the use of affective and cognitive language, the validity of the method is challenged by contextualization problems; for example, it is unable to discern whether positive or negative emotion words pertain to the patient's history or the physicians' own descriptions.

In another recent study, investigators employed natural language processing (NLP) techniques to quantify the use of “n-grams” —that is, clusters of words—to explore changes pre- and post-implementation of open notes (21). Analyzing more than 100,000 notes written by 36 hematology/oncology clinicians, researchers found that, on average, there was no change in n-grams (21). While this method offers a fast method of text-mining at scale, it is unclear how to interpret these changes, or lack of thereof, at a semantic level. Newer research in deep learning for sentiment analysis offers the potential to look beyond clusters of words and attempt to understand the representation of sentences—though still an area of active research (22). Indeed, the utility of machine learning and NLP techniques in analyzing clinical notes is currently limited although evolving, and such methods cannot yet reliably decipher meaningful changes in documentation—for example, whether more or simpler explanations are offered for medical treatments; or whether clinicians offered more supportive care (23). The best analytical methods are only as good as the data that they are trained against and efforts toward improved classification of notes will require unique collaboration with both patients and clinicians offering insight into their meaning, intentions, and reactions.

A range of existing software packages, however, can be employed to compare the length and comprehensibility of notes before and after patient access. For example, computer programs that use validated metrics such as the Flesch–Kincaid reading scale can track the number of words per sentence, word length, and the number of syllables per word to obtain meaningful measures of readability. This may provide a useful route to compare differences in documentation pre- and post- open notes. To complement this approach and pending further advancements in NLP, qualitative research may also provide a valuable method to help assess objective changes in the socio-emotional tone and content of notes, and in how physicians list differential diagnoses. While its scale and speed is considerably restricted, traditional thematic analysis may help to provide deeper insights about potential documentation changes after patient access to notes.

## Conclusions and Recommendations

Patients have a right to access their medical information (see Boxes 1, 2). Open notes are increasingly common, and will continue to grow. As more care continues to be delivered via telemedicine because of COVID, access to notes may help patients better adjust to this new care delivery format (24, 25). While innovation brings about new benefits it also invites unforeseen challenges. Most patients report feeling empowered by online access to their clinical notes but further research is needed to investigate how the practice might influence documentation practices, and the consequences for patients and other health professionals.

Box 1 **Key Messages**

- Online access to clinical notes via patient portals (“open notes”) is growing, and patients', and clinicians' experiences of the practice are generally positive.
- With the knowledge patients might read their clinical notes, some clinicians report changes to documentation practices including: removing language perceived as critical, adding collaborative or encouraging wording, and being less detailed in notes.
- Further research is needed to explore objective changes to documentation as a result of open notes including how clinicians might optimize this communication tool to benefit patients and health professionals.

Box 2 **Key questions and findings**

**What is already known about this topic?**

➢ Worldwide, increasing numbers of patients can access their clinical notes via online patient portals (“open notes”).
➢ In extensive surveys patients describe benefits of open notes. Many report that the practice encourages engagement, recall and understanding of care plans, and strengthens patient-clinician relations.
➢ While there is some variation between medical specialties and between countries, after implementing open notes most clinicians are also positive about the practice.
➢ With the knowledge that patients might read their clinical notes, clinicians do report adjusting their documentation practices including: avoiding language perceived to be critical of patients, being less detailed in notes, and changing how they document differential diagnose

**What are the new findings?**

➢ Limited research has been conducted into assessing objective changes to the content and tone of clinical notes as a result of patient access.
➢ Documentation changes may be positive, enhancing patient understanding and providing reassurance and support. However, some changes may interfere with clinical reasoning.
➢ Further research is needed to develop objective measures of documentation change and to explore how clinicians might optimize clinical notes to improve patients' experiences and outcomes.
➢ As the practice of open notes continues to grow, clinicians may need training in how to preserve the traditional functions of medical documentation while maximizing the potential new benefits of this communication tool.

In the meantime, we recommend that supportive, empathic, and encouraging wording in clinical notes may help to strengthen patient-doctor relationships (26, 27). Such language may have the potential to improve treatment adherence, health engagement, and outcomes for patients. We also recommend that open notes might be optimized to communicate in clear and understandable language the reasons and rationales for tests and treatments. In contrast, we strongly oppose changes that may undermine clinical reasoning, including omitting of key medical information and differential diagnoses. Notwithstanding, modifications in medical documentation may be feasible while maintaining the original function of notes as an accurate and detailed aide memoir for physicians. For example, it may be possible to list differential diagnoses in ways that are fully transparent and honest (and therefore of utility among health professionals) but also reassuring for patients. Other strategies—such as automatic annotation of notes via tooltips—might help to facilitate patient understanding of medical terminology without burdening clinician workflow (28).

As with all new technologies, changes in work practice can be more challenging to implement than the technology itself. In a recent survey, only a quarter of dermatopathologists reported that if their notes were accessible they would need specialized training in how to communicate with patients (14). Going further, we suggest that in the new era of open notes, it is imperative that all clinicians are trained in how to preserve the traditional functions of medical notes, and in maximizing the potential new benefits of this communication tool. Preliminary evidence from web-based clinician training programs suggests that this is achievable (29). Tracking changes in clinical documentation will be key to assess the impact of clinician training, and to evaluate how modifications to clinical notes influence patients' experiences and clinical outcomes.

Finally, by offering patients online access to their clinical notes, the documentation may be more correctly viewed as co-owned by patients and clinicians. Looking ahead, however, it is conceivable that this balance will shift with patients taking even greater control, and potentially becoming the outright owners of their clinical records (30). Indeed, developments are already underway for patients to co-generate medical documentation by setting pre-visit agendas, and providing feedback on their care (25, 31). Interactive notes—so-called “OurNotes”—could offer several important benefits to the quality of documentation, and as a result, patient care. This innovation allows patients to report on their concerns, and describe their health status since their last visit. In addition, co-produced notes offer a more direct opportunity for patients to point out factual inaccuracies in documentation, and to document subjective effects, and side-effects of treatments. Such advancements in clinical documentation may help to close the feedback loop on care (32).

## Author's Note

CB, Ph.D., (guarantor) is an interdisciplinary researcher and philosopher of medicine based at OpenNotes, Beth Israel Deaconess Medical Center who investigates the ethical considerations of sharing clinical notes with patients. JT, M. D.MBI., is an assistant professor and psychiatrist and Director of Digital Psychiatry at Beth Israel Deaconess Medical Center. His research focus on digital mental health and clinical work in augmenting care with smartphone interventions. MH, Ph.D., is an associate professor in health informatics and senior lecturer in implementation science at Uppsala University, Sweden, and a Keane OpenNotes scholar at Beth Israel Deaconess Medical Centre. MH is a member of the Swedish research network DOME that studies the implementation and use of open notes in Sweden, and she chairs a European Working Group on Citizen Health Data.

## Author Contributions

CB, MH, and JT conceived manuscript and revised drafts. CB wrote first draft. All authors contributed to the article and approved the submitted version.

## Conflict of Interest

The authors declare that the research was conducted in the absence of any commercial or financial relationships that could be construed as a potential conflict of interest.
